# Aging effects on discrimination learning, logical reasoning and memory in pet dogs

**DOI:** 10.1007/s11357-015-9866-x

**Published:** 2016-01-04

**Authors:** Lisa J. Wallis, Zsófia Virányi, Corsin A. Müller, Samuel Serisier, Ludwig Huber, Friederike Range

**Affiliations:** 1 Clever Dog Lab, Messerli Research Institute, University of Veterinary Medicine Vienna, Medical University of Vienna, University of Vienna, Veterinärplatz 1, 1210 Vienna, Austria; 2 Department of Cognitive Biology, University of Vienna, Vienna, Austria; 3 Royal Canin Research Center, Aimargues, France

**Keywords:** Touchscreen, Learning, Flexibility, Reasoning by exclusion, Logical reasoning, Working memory, Long term memory, Dog

## Abstract

In laboratory dogs, aging leads to a decline in various cognitive domains such as learning, memory and behavioural flexibility. However, much less is known about aging in pet dogs, i.e. dogs that are exposed to different home environments by their caregivers. We used tasks on a touchscreen apparatus to detect differences in various cognitive functions across pet Border Collies aged from 5 months to 13 years. Ninety-five dogs were divided into five age groups and tested in four tasks: (1) underwater photo versus drawing discrimination, (2) clip art picture discrimination, (3) inferential reasoning by exclusion and (4) a memory test with a retention interval of 6 months. The tasks were designed to test three cognitive abilities: visual discrimination learning, logical reasoning and memory. The total number of sessions to reach criterion and the number of correction trials needed in the two discrimination tasks were compared across age groups. The results showed that both measures increased linearly with age, with dogs aged over 13 years displaying slower learning and reduced flexibility in comparison to younger dogs. Inferential reasoning ability increased with age, but less than 10 % of dogs showed patterns of choice consistent with inference by exclusion. No age effect was found in the long-term memory test. In conclusion, the discrimination learning tests used are suitable to detect cognitive aging in pet dogs, which can serve as a basis for comparison to help diagnose cognition-related problems and as a tool to assist with the development of treatments to delay cognitive decline.

**Electronic supplementary material:**

The online version of this article (doi:10.1007/s11357-015-9866-x) contains supplementary material, which is available to authorized users.

The development and aging of cognitive processes such as learning, memory and logical reasoning and their interactions with genetic, environmental and social factors have so far almost exclusively been studied in humans (Baltes 1987; Craik and Bialystok 2006). Learning and memory are basic processes, which are essential for the acquisition of knowledge, and furthermore allow an individual to apply knowledge in novel situations through logical reasoning. These basic cognitive abilities are known to change over the lifespan, increasing rapidly from infancy to young adulthood and then, depending on the specific ability, are either improved (as is the case for knowledge formation), maintained or declined in old age (Baltes 1987; Pearce 2008).

Cognitive processes are regulated by executive functions comprising selective attention, working memory, flexibility and inhibition, some of which have also been found to be particularly sensitive to aging (Cepeda et al. 2001; Clark et al. 2006; Manrique and Call 2015; Rapp 1990; Tapp et al. 2003a, b; Wallis et al. 2014). There are remarkably few studies in humans or animals which detail the changes in these specific cognitive processes and their regulation by executive processes over the course of the entire lifespan, as cognitive development and aging are frequently disassociated. Previous studies in humans using cognitive batteries showed that learning and logical reasoning increase rapidly from infancy to young adulthood and then decline steadily (Craik and Bialystok 2006; Moshman 2004) and that long-term memory increases into the fifth and sixth decades of life and only shows very gradual decline thereafter (Brickman and Stern 2010).

Learning ability is often measured in human and animal studies using one specific type of learning called discrimination learning. Discrimination learning protocols generally utilise a two-choice procedure, where two stimuli are presented, but only one of them leads to a reward. Since the stimuli are presented simultaneously, parallel processing is necessary. The subject is required to attend to a target stimulus, while ignoring or avoiding ‘distractor’ information (Julesz and Schumer 1981). Selection of the target stimulus results in positive reinforcement, which causes an increase in the frequency of the choice of this stimulus (Mell et al. 2005). Deficits in simultaneous processing of stimuli increase with age in humans and animals, due to decreases in processing speed, reduced cognitive resources and an inability to ignore distracting information (Baddeley et al. 2001; Costello et al. 2010; Lavie 1995; Snigdha et al. 2012). Age-related impairments in learning are shown by an increase in the number of trials necessary to reach a learning criterion as well as an increase in perseverative responding, which is defined as the repetition of a particular response, such as selection of a particular stimulus, due to an inability to adapt to external feedback of right and wrong. Perseverative responding may be a sign of reduced cognitive flexibility, which is the ability to adjust thinking or attention in response to changing goals and/or environmental stimuli (Scott 1962).

Another form of learning is learning by exclusion, a type of logical reasoning defined as the selection of the correct alternative by logically excluding other potential alternatives (Call 2006). Human children are known to learn by exclusion, which develops from the age of 2 years (Heibeck and Markman 1987; Horst and Samuelson 2008; Spiegel and Halberda 2011). Since children as young as 2 years are able to make simple inferences by exclusion, this ability likely depends on simple associative learning mechanisms and therefore can also be found in animals, based on previous positive findings (Aust et al. 2008; Call 2006; Herman et al. 1984; Kaminski et al. 2004; Kastak and Schusterman 2002; Pilley and Reid 2011). For example, Aust et al. (2008) found evidence of reasoning by exclusion in pet dogs using a touchscreen procedure. Additionally, Kaminski et al. (2004) found that a Border Collie had the ability to acquire the relation between a word and the object that the word refers to (the referent) and that it could also infer the referent of new words by exclusion learning and retain this knowledge over time. However, dogs’ preference for novelty could also explain Kaminski et al.’s results (see Kaulfuss and Mills (2008)). The study of Pilley and Reid (2011) on another Border Collie ruled out any influence of novelty preference, by including baseline novelty preference measurements (but see Griebel and Oller (2012) for an alternative conclusion on the dogs’ performance).

Currently, there are no studies in non-human animals detailing how the ability to reason by exclusion changes with age over the lifespan. Studies in humans, however, have demonstrated that logical reasoning ability is closely related to an individual’s working memory capacity, which is limited in complex tasks (Kyllonen and Christal 1990; Süß et al. 2002). Working memory capacity can severely limit reasoning abilities particularly in tasks where time limits are implemented (Chuderski 2013). Moreover, in order to reach learning criterions in complex discriminations and learning by exclusion tasks, long-term memory is required to store information such as positive and negative stimulus associations in discrimination learning or the correct labelling of a new word or object in exclusion tasks. While working memory and logical reasoning ability decline with old age (Borella et al. 2008; Brockmole and Logie 2013; De Luca et al. 2003; Lee et al. 2005; Sander et al. 2012), long-term memory shows very little decline when comparing younger and older adults (Brickman and Stern 2010).

Learning and memory have been extensively studied in laboratory dogs which are considered to be a good animal model for human aging and Alzheimer’s disease, since they develop similar age-related neuropathologies as humans, as well as a similar decline in their measures of sensorimotor ability, selective attention, learning, short-term memory and executive function with age (Adams et al. 2000a, b; Head et al. 1995; Head et al. 2000; Landsberg et al. 2003; Milgram et al. 1994; Tapp et al. 2003a, b; Wallis et al. 2014). For example, like humans, dogs’ selective visual attention and discrimination learning is sensitive to aging in some tasks (Milgram et al. 2002; Snigdha et al. 2012), whereas in other tasks discrimination learning was not affected by age (egocentric spatial discrimination, Christie et al. 2005; object discrimination learning, Milgram et al. 1994). This inconsistency in laboratory dogs is likely explained by the level of difficulty of the task which influences whether an age effect is detected or not (Adams et al. 2000a, b; Head et al. 1998; Milgram et al. 1994). Previous research has also shown that older dogs tend to show perseverative responding in complex discrimination learning tasks similarly to humans (Grant and Berg 1948; Mell et al. 2005; Tapp et al. 2003b).

Few studies have addressed how long dogs are able to remember previously learnt discriminations, which is a measure of long-term memory. Araujo et al. (2005) tested laboratory beagles in a working memory task and found a significant decline with age. In contrast, their performance remained stable after a 2-year break period in previously learned discriminations. Therefore, working memory capacity in dogs declines with age, whereas long-term memories are more resistant to aging, which reflects similarities to humans (Adams et al. 2000a, b; Fiset et al. 2003; Fiset 2007; Salvin et al. 2011; Tapp et al. 2003b).

Most research projects have relied on laboratory-kept beagles to examine age-related cognitive changes. One advantage of utilising pet dogs living with human families is that we are able to examine the development and aging of cognition under the influence of the human living environment. This environment is likely to be more enriching and stimulating than that found in laboratory-housed beagles and thus may provide a greater level of resistance to the effects of aging (Milgram et al. 2005). There are few studies which have examined age-dependent losses in learning and memory in companion dogs (González-Martínez Á et al. 2013; Mongillo et al. 2013; Salvin et al. 2011). Such studies are crucial for the development of objective diagnostic procedures to enable the accurate diagnosis of canine cognitive dysfunction syndrome (age-related non-normal cognitive decline) and to quantify normal successful aging in pet dogs outside a laboratory setting.

The use of the touchscreen apparatus allows the design and implementation of non-verbal standardised tasks which can be utilised to examine cognitive functioning such as individual learning abilities, memory and logical reasoning in non-human animals and permits comparisons with humans and across species (Spinelli et al. 2004; Steurer et al. 2012). Computerisation results in the elimination of social cuing and increases/maintains the motivation to work in the subjects (Range et al. 2008). The touchscreen can be used to establish baseline measures of cognitive aging associated with normal aging, which has so far only been utilised in humans (Clark et al. 2006), laboratory-housed non-human primates (Joly et al. 2014; Nagahara et al. 2010) and rodents (Bussey et al. 2008).

Accordingly, the goals of the present study were to test the effect of aging on discrimination learning, reasoning by exclusion and memory in a cross-sectional sample of pet dogs ranging in age from 5 months to 13 years, in order to determine when dogs cognitively mature and when cognitive decline begins. After receiving pre-training on how to work on a touchscreen, the dogs were tested in four tasks: (1) underwater photo versus drawing discrimination consisting of six stimuli, (2) clip art picture discrimination consisting of eight stimuli (which were also used as a training for the next task on inferential reasoning by exclusion), (3) inferential reasoning by exclusion testing, and (4) a memory test on the clip art picture discrimination (task 2) performed after a 6-month break from the touchscreen. Two discrimination tasks were utilised which differed not only in the types and number of stimuli used but also in their difficulty level. In the first discrimination (underwater photo vs. drawing), the positive and negative class was composed of highly similar members with large inter-class and small intra-class differences, whereas the more difficult second discrimination (clip art pictures) had equal inter-class and intra-class differences. Based on previous studies in laboratory dogs, we predicted that dogs’ learning ability will decrease with age and perseverative responding will increase (Milgram et al. 2002; Snigdha et al. 2012; Tapp et al. 2003a). Long-term memory was predicted to remain stable with age (Araujo et al. 2005), and finally, based on information from the human literature, the ability to make inferences by exclusion was predicted to peak in young adulthood and decline thereafter (Moshman 2004), in conjunction with dogs’ working memory ability (Tapp et al. 2003b).

## Methods

### Subjects

Ninety-five pet dogs ranging in age from 5 months to 13 years and 10 months were recruited to participate in the study (Table 1). All dogs were from one breed, the Border Collie, in order to exclude the effects of different developmental and aging speeds of different breeds. The subjects were split into five age groups according to Siegal and Barlough (1995), which aimed to reflect the developmental periods in the Border Collie (late puppyhood, adolescence, early adulthood, middle age and late adulthood which included senior and geriatric).

**Table 1:** Age, sex and neuter status of subjects

Age group	Life stage	Age in months	Mean + SD age in years	Male (neutered)	Female (neutered)	Total
Group 1	Late puppyhood	5–12	0.68 + 0.16	7 (0)	13 (1)	20
Group 2	Adolescence	>12–24	1.39 + 0.24	10 (1)	12 (2)	22
Group 3	Early adulthood	>24–36	2.42 + 0.30	7 (3)	14 (5)	21
Group 4	Middle age	>36–72	4.41 + 0.89	5 (2)	13 (6)	18
Group 5	Late adulthood	>72	8.61 + 2.10	5 (3)	9 (9)	14
Total				34 (9)	61 (23)	95

### Apparatus

**Fig. 1:**
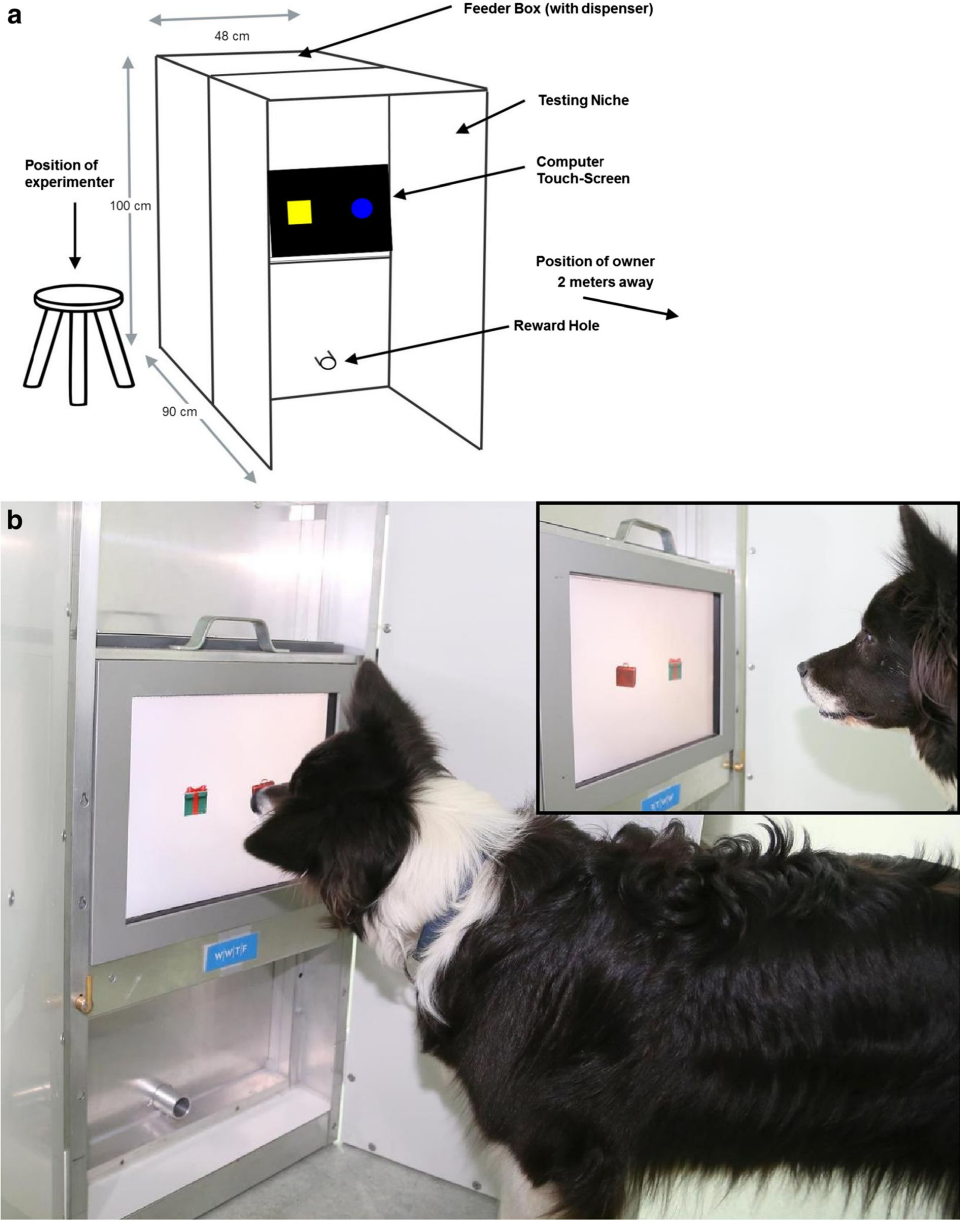
**a** Schematic drawing of the apparatus and **b** photograph of a dog working in the testing niche with one side open

Testing was conducted in a room (3 × 4 m) at the Clever Dog Lab in Vienna, Austria. The test apparatus consisted of a closed rectangular box containing the food pellet dispenser (feeder box; 48 × 100 × 60 cm (w × h × d)) and an adjacent testing niche (48 × 100 × 30 cm) where the touchscreen was located along the top back wall (Fig. 1). Dogs were tested in the testing niche, which allowed subjects to reach the touchscreen whilst their vision was shielded to avoid potential distractions from the side or above, thus minimising human influence on the dogs’ performance. Inside the testing niche, a 15″ TFT 600 × 800 pixel resolution computer screen was mounted behind an infrared touchframe (Carroll Touch, Round Rock, TX, USA; 32 vertical × 42 horizontal resolution (Aust et al. 2008; Huber et al. 2005; Range et al. 2008; Steurer et al. 2012)). A small hole beneath the touchscreen allowed commercial dog food pellets to be automatically dispensed in order to administer reinforcement for correct choices. The presentation of the stimuli and the release of the reward were controlled by a microcomputer interfaced through a digital input–output board. The owner and the experimenter were present during the testing but were prevented from viewing the stimuli by the walls of the testing niche (see Fig. 1a for owner and experimenter locations).

### Procedure

The touchscreen training and testing procedures consisted of two pre-training steps (an approach training and a simple geometric form discrimination) and four tasks: a ‘categorical’ discrimination (underwater photographs and drawings; task 1), a clip art picture discrimination (the training phase of the inferential reasoning by exclusion tests; task 2), inferential reasoning by exclusion testing (previously reported in Aust et al. 2008; task 3) and finally task 4: a memory test after a 6-month break from the touchscreen consisting of a repetition of task 2 (clip art picture discrimination/inference by exclusion training).

### Touchscreen pre-training

#### Approach training

Dogs visited the lab once a week and participated in three to four sessions (each session consisted of 30 to 32 individual trials), over a half-hour period, with short breaks in between sessions. Dogs were trained to touch the monitor with their nose using a clicker-aided shaping procedure. A stimulus, either a circle or a square, appeared in random locations on a black screen. If the dogs touched the stimulus with their nose, the infrared light grid was interrupted, which triggered an acoustic signal and delivery of a food treat. After the dog became familiar with the action of touching the stimulus and receiving the food reward via the automatic feeder (without help from the experimenter), the simple geometrical form discrimination was initiated.

#### Geometric form discrimination

**Fig. 2:**
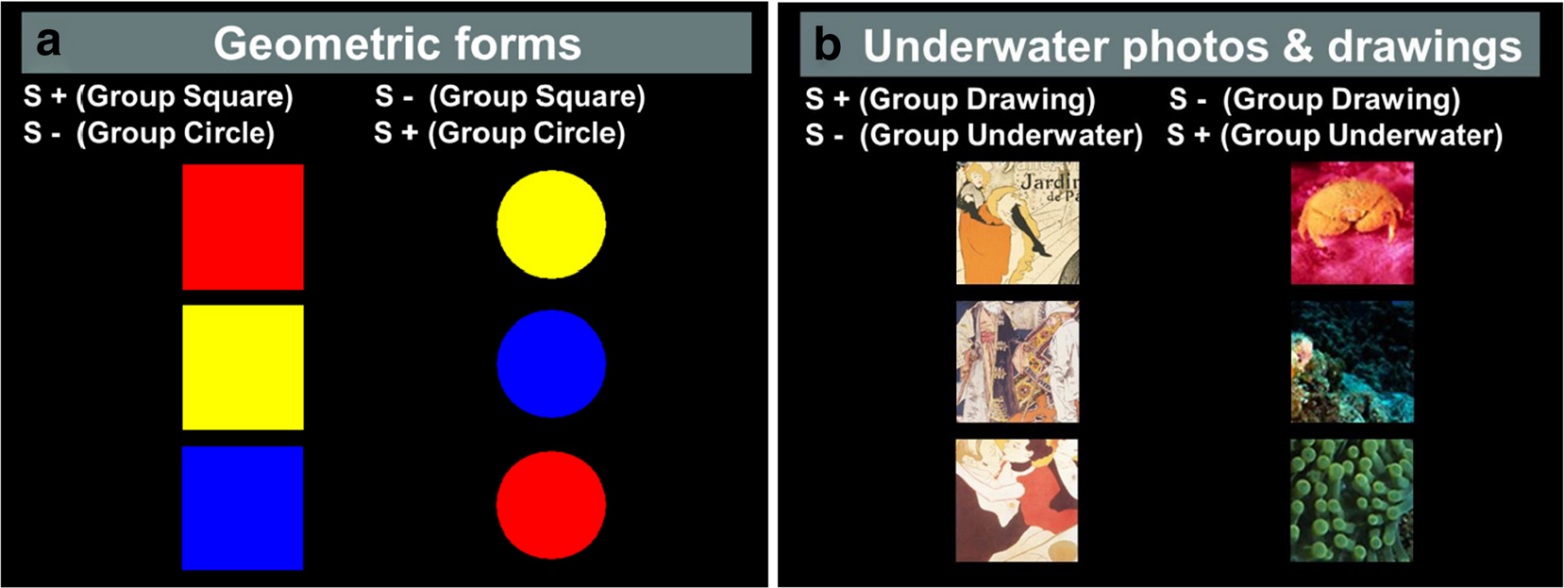
Training stimuli for the **a** geometric form and **b** underwater photo and drawing discriminations

In this task, the subjects were shown a square and a circle side by side. Both stimuli were varied in colour between trials (red, yellow or blue, Fig. 2a). The dogs were assigned to two groups balanced for age group and sex. Group ‘square’ was rewarded for touching the square; group ‘circle’ was rewarded for touching the circle. A forced two choice procedure was utilised, where the two shapes were presented simultaneously on a black background in fixed positions on the screen (at the animal’s eye level, one appearing left of the middle, and the other right, Fig. 1). Each trial was composed of one positive stimulus (S+) and one negative stimulus (S−), which were positioned randomly from trial to trial (left/right). Each session consisted of 30 trials. When the positive stimulus was selected, both stimuli disappeared, a short tone was emitted by the computer, and a food reward was provided. If the wrong stimulus was touched (S−), both stimuli disappeared, a short buzz sounded, and a red screen was presented for 3 s. In this case, a correction trial was immediately initiated: the stimuli of the previous trial were presented again in the same positions. A correct choice terminated the trial and resulted in reward and presentation of a new trial. After each trial (except correction trials), an inter-trial interval of 2 s was initiated (an empty black background was presented). The learning criterion was set at ≥20 correct first choices in 30 trials (66.7 %) in four out of five consecutive sessions. At this early stage in the training, the experimenter often needed to give dogs extra help in sessions, for example, verbal encouragement to approach the screen and touch, and occasional pointing. Therefore, the results from this test are presented only in the supplementary materials (Table S1).

### Touchscreen testing

#### Task 1: underwater photos and drawings discrimination

Once the criterion for the geometric form task was reached, the dogs were transferred to a second discrimination training, involving three underwater photographs, which had to be distinguished from three drawings (two of which were taken from posters by Toulouse-Lautrec; Fig. 2b). The dogs were assigned to two groups balanced for age group and sex. Group ‘drawing’ was rewarded for touching the drawing and group ‘underwater’ was rewarded for touching the underwater photograph. In each trial, one of the three S+ was randomly coupled side by side with one of the three S−. The procedure and learning criterion were the same as for the geometric form discrimination.

#### Task 2: clip art picture discrimination (training for task 3: inferential reasoning by exclusion)

**Fig. 3:**
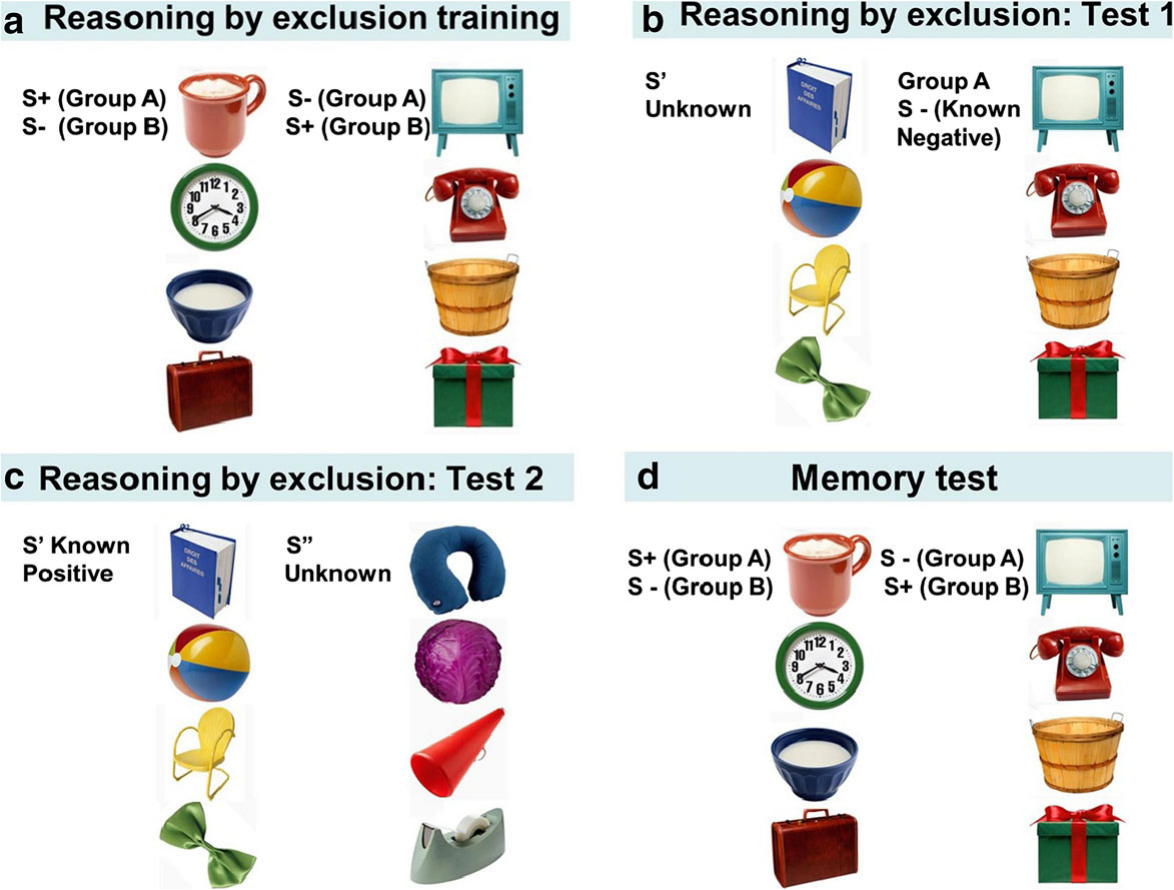
**a** Reason by exclusion training stimuli, **b** test 1 stimuli, **c** test 2 stimuli, and **d** memory test stimuli

Once the dogs had completed the underwater photos and drawing discrimination, they began the training for the inference by exclusion tests. Dogs were again split into two groups (Group ‘A’ and Group ‘B’) balanced for age group and sex. The dogs were trained to discriminate four S+ and four S− stimuli (Fig. 3a), this time presented on a white background. Once again, the forced two-choice procedure was utilised. The stimuli were coloured clip art pictures obtained from the internet and were grouped within the two sets by avoiding similarities in colour, form or function. The clip art stimuli were the same as those used by Aust et al. in the 2008 study. Each session consisted of 32 trials and contained each of the 16 possible S+/S− pairings twice per session. All dogs were required to reach two learning criteria: a first learning criterion of ≥28 correct first choices (87.5 %) in two consecutive sessions and a final learning criterion of ≥28 correct first choices in five of seven consecutive sessions before beginning testing. Thirteen dogs which were tested prior to 2010 were trained on a 100 % reward ratio. For the remaining 72 dogs, the reward ratio was reduced stepwise to 75 % (for explanations of the rationale for a change in methodology, please see Supplementary Material: Reward ratio reduction). The unrewarded trials in the training served to familiarise the dogs with the testing procedure, which included up to eight unrewarded test trials in each session. Initially, training sessions for these dogs included four trials that were not rewarded: i.e., the first choice of any of the two stimuli terminated the trial without any acoustic or visual feedback, correction trial or reward. The first learning criterion was utilised (≥28 correct first choices in two consecutive sessions), and once dogs reached this criterion, the reward ratio was further reduced to six unrewarded trials per session. The same learning criterion was applied again, after which a final training phase with a 75 % reward ratio (eight unrewarded trials) was applied. The final learning criterion was used for this phase (≥28 correct first choices in five of seven consecutive sessions), the same criterion as was used for the 13 dogs originally tested with the 100 % reward ratio.

#### Task 3: inferential reasoning by exclusion

- Test 1: Test sessions consisted of 28 training trials with four randomly interspersed test trials (a total of 32 trials per session). The test trials contained four known S− from the training trials, which were paired with four novel stimuli (Fig. 3b). The new stimuli (S’) replaced the S+ from the training. Each of the 16 test combinations were shown twice, once in cycle 1 (sessions 1–4) and once in cycle 2 (sessions 5–8). Subjects which choose by exclusion should choose S’ due to inference of positive class membership; i.e., by assuming, there is always a member of the positive class and by excluding S− due to its formed association with the negative class. But dogs which choose according to novelty (neophilia) or avoidance of S− should also choose S’. In contrast, subjects which choose by familiarity should prefer S−. Dogs which chose S’ in ≥22 out of a total of 32 test trials proceeded directly to test 2.
- Test 2: In order to confirm that dogs chose by exclusion, an additional test was run to exclude that dogs chose based on novelty or avoidance of S−. The subjects were again tested with one of the four S’ paired with a known S− (same as test 1, Fig. 3b, hereafter known as the test 1 refresher) to refresh their memory, and then in one of the next two to three trials, they were presented with the same S’ paired with one of four novel alternative stimuli S” (Fig. 3c). If dogs chose by inference by exclusion, they would choose S’ when paired with the known negative (in tests 1 and 2 (in the test 1 refresher)) and also choose S’ when S’ was paired with the novel S”. Subjects which showed a preference for S’ in test 1 due to neophilia would now prefer the more novel S” over S’ (novelty preference). Subjects which avoided S− in test 1 without making any inferences about the positive association of S’ would choose randomly in test 2, showing no preferences.
  In each session in test 2, there were eight non-rewarded trials (four test 1 refresher and four test 2 trials) interspersed within 24 training trials (32 trials in total per session). Each of the 16 test combinations (four known S’ from test 1, paired with four novel stimuli (S”)) were again shown twice, once in cycle 1 (sessions 1–4) and once in cycle 2 (sessions 5–8).
  For each test 2 trial, dogs were scored as choosing by inference by exclusion if they firstly chose S’ when paired with the known negative (test 1 refresher) and also chose S’ in the subsequent trial when S’ was paired with the novel S” (test 2 trial). Over the entire test 2, dogs were scored as choosing by inference by exclusion above chance if they chose by exclusion in 13 or more out of the possible 32 test trials (binomial test, chance level = 0.25, *p* = 0.016 (chance level reflects the four possible choice combinations of test 1 refresher, and test 2 trial; S’ and S’, S’ and S”, S− and S’, and finally S− and S”)).

#### Task 4: memory test

After completing the tests, all dogs had a minimum of 6 months break before they were invited back to participate in a memory test consisting of a repetition of task 2 (clip art picture discrimination/inference by exclusion training), up to the final criterion of ≥28 correct first choices (87.5 %) in five of seven consecutive sessions (Fig. 3d). Dogs, which had been trained on the 75 % reward ratio repeated the task at the 75 % reward ratio, and dogs, which were trained on the 100 % reward ratio, repeated the task at the 100 % reward ratio. The total number of correct choices in the first session of the memory test was used as a measure of memory ability.

### Data analysis

Statistical analyses were performed in R-3.0.1 (R Core Team 2013). Separate statistical models were calculated first with age as a continuous variable (we tested for linear and quadratic relationships) and then with age as a categorical variable to look for specific differences between age groups. Results are presented as mean ± standard deviation unless otherwise indicated.

In the geometric form, underwater photo and drawing discrimination and the clip art picture discrimination, we used the total number of sessions needed to reach criterion minus the minimum number of sessions needed to reach the criterion of each discrimination (in order to fulfill the assumptions for Poisson distribution) and the total number of correction trials as measures of learning speed and behavioural flexibility. In the clip art picture discrimination, the number of sessions needed to reach the first criterion of ≥28 correct first choices in two consecutive sessions in both the 100 % rewarded and the reduced reward groups was used to allow learning speed to be assessed for the different reward ratios. The proportion of test trial choices of S’ in test 1 and the proportion of test trials where dogs chose based on inference by exclusion (in the repetition of S’ paired with S− and the new S” paired with S’) in test 2 were calculated as two separate variables to describe the logical reasoning strategies of the dogs. Finally, the total number of correct choices in the first session of the memory test was used as a measure of memory ability.

Data were analysed using generalised linear models and generalised linear mixed models, with age, stimulus group, sex and neuter status included as fixed effects. In the inference by exclusion training and test 1, we also examined the effect of the type of reward ratio (100 % reward or reduced reward). We included the two-way interaction between stimulus group and age to test whether age effects differed between stimulus groups. When examining the proportion of test trial choices of S’ in test 1 and proportion of test trials where dogs chose based on inference by exclusion in test 2, we also checked whether the dogs’ performance changed from cycle 1 to cycle 2. The full models can be found in the Supplementary Materials (geometric forms discrimination (Table S1), underwater photos and drawings discrimination (Table S2), clip art picture discrimination (Table S3), inferential reasoning by exclusion Test 1 (Table S4), inferential reasoning by exclusion Test 2 (Table S5), and memory test (Table S6)). Non-significant predictors (*p* > 0.05) were then removed from the models and are not reported in the “Results” section. According to the distribution of the response variables, models with negative binomial error structure and log link function (Venables and Ripley 2002) were used for the number of sessions to criterion and the total number of correction trials, as well as models with binomial error structure and logit link function for the proportion of choices of S’ in test 1 and test 2 and the proportion of correct first choices in the memory test. When analysing data including multiple data points per subject, dog identity was included as a random factor in the model. Plots of residuals and Cook’s distance were examined for outliers. Since none of the data points exceeded Cook’s distance of 1, no outliers needed to be excluded.

## Results

### Task 1: underwater photo and drawing discrimination

**Fig. 4:**
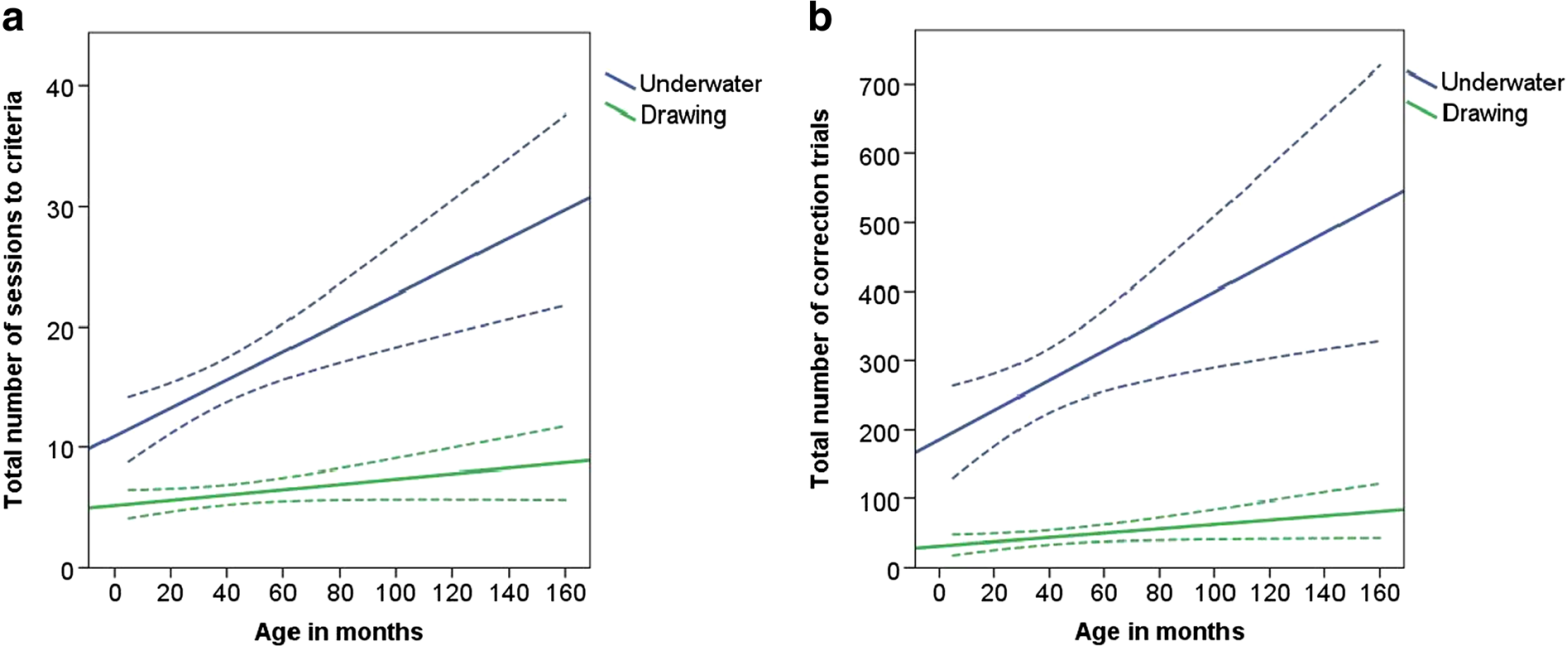
*Line graph* showing the linear relationship between age in months and **a** number of sessions to criterion and **b** number of correction trials, shown separately for dogs that were rewarded for choosing the underwater pictures and for dogs rewarded for choosing the drawings (with 95 % confidence intervals (*dotted lines*))

Of the 95 dogs which began testing with the geometric form discrimination, 93 passed the learning criterion for the underwater photos and drawing discrimination within 35 sessions. The number of sessions to criterion increased linearly with age in months (Table 2: model 1, Fig. 4a). The subsequent age group analysis revealed that age groups 4 and 5 took significantly more sessions to reach criterion compared to age group 1 (model 2). Dogs in the drawing group completed the task in significantly fewer sessions than dogs in the underwater group, reflecting a difference in task difficulty (Fig. 4a).

**Table 2:** Negative binomial generalised linear models showing the direction of effects and the significance level of the terms in the underwater photos and drawings discrimination

Response variable	Model	Minimal model	Average effect	SE	Wald statistic	*z*	*p* value
Number of sessions to criterion	Model 1	Stimulus group: underwater	1.3841	0.1389	68.704		**<0.001**
		Age in months	0.0072	0.0018	14.224		**<0.001**
	Model 2	Age group			14.627		**0.006**
		Age group 2	0.0109	0.1969		0.055	0.956
		Age group 3	0.1200	0.2025		0.593	0.553
		Age group 4	0.4832	0.1937		2.495	**0.013**
		Age group 5	0.6104	0.2121		2.877	**0.004**
Number of correction trials	Model 3	Stimulus group: Underwater	1.7887	0.1470	88.076		**<0.001**
		Age in months	0.0067	0.0022	9.584		**0.002**
	Model 4	Age group			11.181		**0.025**
		Age group 2	−0.0631	0.2135		−0.295	0.768
		Age group 3	0.3723	0.2155		1.728	0.084
		Age group 4	0.4144	0.2151		1.927	0.054
		Age group 5	0.5741	0.2412		2.383	**0.017**

*Z* tests indicate which age groups differ from age group 1 in the respective analysis. Bold numbers indicate significant values at *p* = ≤0.05

The total number of correction trials also increased linearly with age in months (Table 2: model 3, Fig. 4b). Age group 5 needed significantly more correction trials compared to age group 1 (model 4). Dogs in the underwater group had significantly more correction trials than dogs in the drawing group, furthermore supporting the difference in task difficulty (Fig. 4b).

### Task 2: clip art picture discrimination (training for task 3: inferential reasoning by exclusion)

**Fig. 5:**
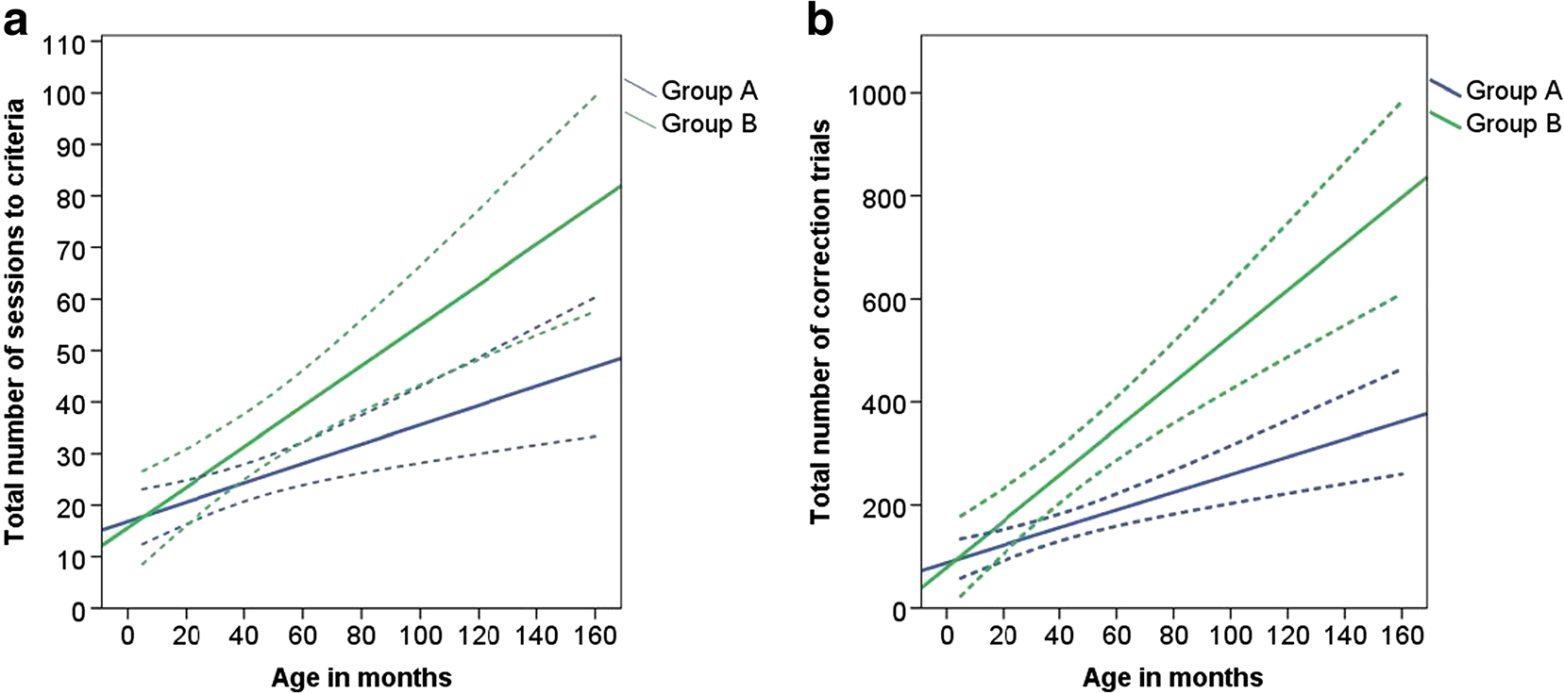
*Line graph* showing the linear relationship between age in months and **a** number of sessions to criterion and **b** number of correction trials, separately for groups A and B (with 95 % confidence intervals (*dotted lines*))

Of the 90 dogs which began the training, 85 passed the first learning criterion of 28 or more correct choices in two consecutive sessions within 7 to 113 sessions. The five dogs (all in age groups 4 and 5), which did not reach the learning criterion, dropped out of the study due to motivation problems. The number of sessions to criterion increased linearly with age in months (Table 3: model 5, Fig. 5a). Age groups 4 and 5 took significantly more sessions to reach criterion compared to age group 1 (model 6). Dogs in group A completed the task in significantly fewer sessions than dogs in group B, reflecting a difference in task difficulty depending on the set of pictures the dogs were rewarded for (Table 3: model 5, Fig. 5a). Male dogs needed more sessions to reach criterion than female dogs (males, 29.03 ± 22.70, *N* = 31: females, 23.48 ± 16.26, *N* = 54; Table 3: model 5). For further results and a discussion of these sex differences, please see Supplementary Materials. Dogs which participated in the reduced reward ratio training took significantly longer to reach the first learning criterion than dogs in the 100 % rewarded group (reduced reward 26.79 ± 18.85, *N* = 72: 100 % rewarded 18.38 ± 18.42, *N* = 13; Table 3: model 5). Please refer to Supplementary Materials for additional results and a discussion of the reward ratio reduction.

**Table 3:** Negative binomial generalised linear models showing the direction of effects and the significance level of the terms in the clip art picture discrimination (training for task 3: inferential reasoning by exclusion)

Response variable	Model	Minimal model	Average effect	SE	Wald statistic	*z*	*p* value
Number of sessions to criterion	Model 5	Age in months	0.0100	0.0017	32.326		**<0.001**
		Stimulus group: B	0.2707	0.1095	5.908		**0.015**
		Sex: male	0.3507	0.1169	8.710		**0.003**
		Reward ratio 90 %	0.3486	0.1545	4.877		**0.027**
	Model 6	Age group			29.633		**<0.001**
		Age group 2	0.0612	0.2046		0.2990	0.765
		Age group 3	0.1162	0.2088		0.5570	0.578
		Age group 4	0.6525	0.2193		2.9750	**0.003**
		Age group 5	0.8879	0.2215		4.0090	**<0.001**
Number of correction trials	Model 7	Age in months	0.0118	0.0019	37.953		**<0.001**
		Stimulus group: B	0.4313	0.1250	11.169		**<0.001**
		Sex: male	0.3184	0.1253	6.296		**0.012**
	Model 8	Age group			32.130		**<0.001**
		Age group 2	0.3174	0.2287		1.388	0.165
		Age group 3	0.2992	0.2338		1.280	0.201
		Age group 4	0.6798	0.2490		2.730	**0.006**
		Age group 5	1.2756	0.2525		5.053	**<0.001**

*Z* tests indicate which age groups differ from age group 1 in the respective analysis. Bold numbers indicate significant values at *p* = ≤0.05

The total number of correction trials increased linearly with age in months (Table 3: model 7, Fig. 5b). Age groups 4 and 5 had significantly more correction trials compared to age group 1 (model 8). Dogs in group B had significantly more correction trials than dogs in group A (Table 3: model 7, Fig. 5b). Male dogs needed more correction trials than female dogs (males = 217.26 ± 159.46, females = 198.52 ± 200.80; Table 3: model 7).

### Task 3: inferential reasoning by exclusion

**Fig. 6:**
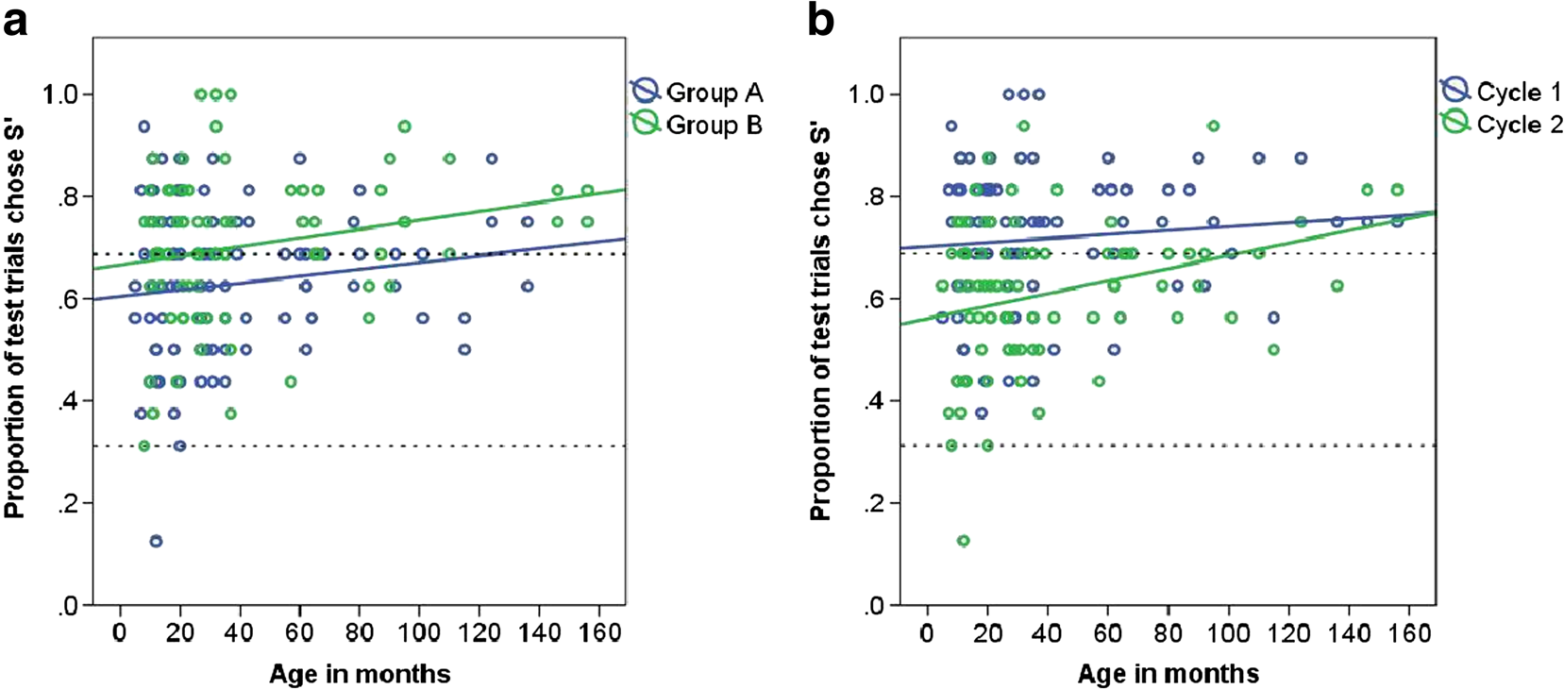
The proportion of test trials in test 1 in which the dog chose S’; **a** group A and group B, and **b** cycle 1 (sessions 1 to 4) and cycle 2 (sessions 5 to 8), and age in months. The *upper dashed line* indicates the levels of performance beyond which preference for S’ was inferred (68.75 %; choice by novelty, avoidance of S− or reasoning by exclusion). The *lower dashed line* indicates the level of performance below which preference for S− was inferred (31.25 %; choice by familiarity)
- Test 2: There was no significant difference between the number of times the dogs chose based on inference by exclusion in cycle 1 and cycle 2, so data were pooled and generalised linear models were applied (see Supplementary Material Table S5: model 11). Seven individuals (17 %) scored above chance, and six of these seven were in group B (Fig. 7). The proportion of test trials in which the dogs chose based on inference by exclusion showed a significant increase with age in months (Table 5: model 12, Fig. 7). Age groups 3, 4 and 5 chose S’ significantly more often compared to age group 1 (model 13). Dogs in group B chose by inference by exclusion in significantly more test trials than dogs in group A (Table 5: model 12, Fig. 7).

- Test 1: Of the 85 dogs which passed the first learning criterion (≥28 correct first choices (87.5 %) in two consecutive sessions), 82 passed the final learning criterion of 28 or more correct choices in five out of seven consecutive sessions and participated in test 1.
  The proportion of test trials in which dogs chose S’ showed a significant increase with age in months (Table 4, Fig. 6). No significant differences between the age groups were detected, however. Dogs in group B chose S’ in significantly more test trials than dogs in group A (Table 4, Fig. 6a). Male dogs showed a tendency to choose S’ more often than females (males, *N* = 30, 0.69 ± 0.02, females, *N* = 52, 0.65 ± 0.01; Table 4). Dogs chose S’ more often in cycle 1 compared to cycle 2 (Table 4, Fig. 6b). When results from cycles 1 and 2 were pooled, 42 (51 %) dogs preferred S’ (choose S’ in 22 or more test trials out of a total of 32) and thus chose based on exclusion (rejection of S− due to its association with the negative class), novelty (selection of S’ due to neophilia) or avoidance of the known negative stimulus (S−) and proceeded to test 2 (apart from one dog which left the study at this stage). The remaining dogs chose at chance level, apart from one individual, which chose based on familiarity.

**Table 4:** Generalised linear mixed model on the proportion of trials chose S’ when paired with a known negative (S−) in test 1 of the inference by exclusion task, showing the direction of effects and the significance level of the terms

Response variable	Model	Minimal model	Average effect	SE	Wald statistic /deviance	*p* value
Proportion of trials chose S’	Model 9	Cycle: cycle 2	−0.4943	0.0839	34.723	**<0.001**
		Stimulus: group B	0.3478	0.1007	11.136	**<0.001**
		Age in months	0.0037	0.0014	6.567	**0.010**
		Sex: male	0.1919	0.0988	3.693	0.055

Bold numbers indicate significant values at *p* = ≤0.05

**Fig. 7:**
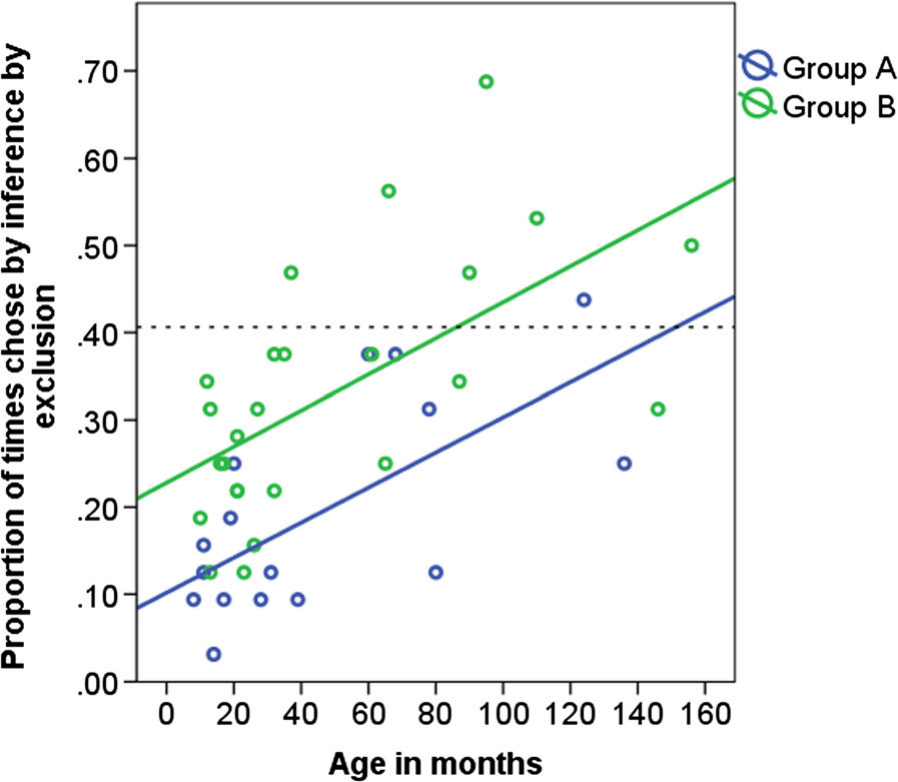
The proportion of times in which the dog chose based on inference by exclusion in group A and group B and age in months in test 2 (cycles 1 and 2 pooled). The *dashed line* indicates the levels of performance beyond which preference for S’ was inferred (40.625 %; reasoning by exclusion)

**Table 5:** Generalised linear model on the proportion of times the dogs’ chose S’ when paired with the known negative (test 1 refresher) and also chose S’ in the subsequent trial when S’ was paired with the novel S” (test 2 trial) in the inference by exclusion task, showing the direction of effects and the significance level of the terms

Response variable	Model	Minimal model	Average effect	SE	Wald statistic /deviance	*z*	*p* value
Proportion of times chose S’ in both test 1 refresher trial and test 2 trial	Model 12	Age in months	0.0099	0.0014	45.538		**<0.001**
		Stimulus: group B	0.7027	0.1367	27.739		**<0.001**
	Model 13	Age group			54.570		**<0.001**
		Age group 2	0.4654	0.2816		1.653	0.094
		Age group 3	0.6387	0.2989		2.137	**0.033**
		Age group 4	1.2223	0.2900		4.215	**<0.001**
		Age group 5	1.3916	0.2788		4.992	**<0.001**
	Model 14	Sessions to criterion	0.0008	0.0029	0.082		0.775
	Model 15	Total no. of correction trials	0.0006	0.0003	4.103		**0.043**

*Z* tests indicate which age groups differ from age group 1 in the respective analysis. Age in months was included in models 12 and 13 to control for age effects. Bold numbers indicate significant values at *p* = ≤0.05
  The proportion of test trials in which dogs chose by exclusion showed a significant increase with the total number of correction trials in the inference by exclusion training (Table 5, model 15) after controlling for age in months. Therefore, regardless of age, dogs which needed more correction trials in the training chose more often using inference by exclusion in test 2.

### Task 4: memory test

Of the 82 dogs which completed the final learning criterion of the inference training, 46 participated in the memory test after a break of at least 6 months. Forty-two of these dogs scored significantly above chance level in the first session (22 or more out of the possible 32 first correct choices (binomial test 22/32 = 0.6875, chance level = 0.5, *p* = 0.050; 81.52 ± 10.10 %). There were no significant effects of age or stimulus group on the proportion of correct first choices in the first session of the memory test (Supplementary Table S6).

## Discussion

The aim of the present study was to examine age effects on visual discrimination learning, inferential reasoning by exclusion and long-term memory in domestic dogs kept as pets. We found a significant effect of age on the number of trials needed to reach criterion (as age increased, discrimination learning ability decreased) and degree of perseveration (the number of correction trials) in the two visual discrimination learning tasks. In contrast, older dogs chose more often by exclusion than younger dogs in the crucial (second) reasoning by exclusion test. Finally, dogs’ long-term memory was maintained into old age, with no difference in performance in any of the age groups after a 6-month break from the touchscreen.

The ability to learn new visual stimulus associations decreased with age as predicted. The youngest dogs aged from 5 months to 1 year needed the lowest number of sessions to complete the criteria, indicating that this age group was already performing at peak performance, and from this age onward, dogs’ learning abilities began to decline. In contrast to the present study, previous studies in non-human animals have found no effect of aging on associative learning in simple object discrimination tasks either in the rhesus macaque (aged from 3 to 34 years; Bachevalier et al. 1991) or in laboratory dogs (aged from 1.5 to 11 years; Milgram et al. 1994). One possible reason for this discrepancy is that, by utilising a higher number of stimuli to be discriminated, we sufficiently increased the difficulty level and thus facilitated the appearance of age effects. This interpretation is also supported by the difference we find between the two stimuli groups both in the drawing and underwater photo discrimination and in the clip art discrimination: If the discrimination seems to be easier for the dogs (‘drawing’; group B), the age differences, although still apparent, are not as pronounced as in the more difficult groups (‘underwater’; group A). However, although age effects were more apparent in the groups with the less preferred stimuli as positive (that is, in the more difficult version of each task), we found no evidence for an interaction between age and stimulus group in any of the discrimination tasks. For a discussion of stimulus preferences in two choice discriminations, please refer to the Supplementary Materials: Stimulus preferences.

Age differences were more pronounced in the clip art picture discrimination than in the drawing and underwater photo discrimination. This difference in effect size may be explained firstly in terms of the number of stimuli to be discriminated (six in the drawing and underwater discrimination and eight in the picture discrimination) and additionally by the fact that the drawing discrimination could be solved more easily by learning a perceptual discrimination rule. All the drawings looked perceptually similar to each other, as did the underwater photographs, but the clip art picture discrimination required that all the stimuli be encoded into memory individually, as there were no perceptual commonalities in the positive or the negative stimuli. Our results are in line with the findings from human studies; age effects can be better detected by more complex tasks (Alvarez and Emory 2006; Mell et al. 2005).

The poorer performance of dogs aged over 3 years in our study could be explained by several possibilities. First, older dogs may suffer from attentional deficits due to reduced processing resources (Snigdha et al. 2012). Additionally, older dogs may use ineffectual strategies in an attempt to solve the discriminations, for example, a stimulus response strategy (such as stimulus preferences or avoidance, as seen when dogs repeatedly make incorrect choices) and/or a positional strategy (side bias), before finally switching to a cognitive strategy. Both stimulus response and positional strategies require less working memory and are therefore less costly than a cognitive strategy (Chan et al. 2002). Unfortunately, we were unable to analyse positional strategies due to limitations in the software program.

Second, younger dogs may have been quicker to utilise the cognitive strategy of forming reward associations for the positive stimuli by utilising working memory and swift-encoding to long-term memory. These younger dogs, assuming that their working memory abilities were good, might have shown more focused selective attention allowing them to quickly pick out the correct stimuli and ignore the negative stimuli (Mongillo et al. 2010; Snigdha et al. 2012; Wallis et al. 2014). In contrast, older dogs have a reduced capacity for working memory (Chan et al. 2002; Tapp et al. 2003b), similarly to other species including humans (Cowan 2001; Matzel and Kolata 2010). Evidence in humans suggests that older individuals with lower working memory capacity may also need to cope with the processing of negative (or distractor) stimuli, which leads to slower learning and the storage of more information in memory than younger individuals with high working memory capacity (Konstantinou et al. 2014; Vogel et al. 2005).

Third, an important non-cognitive factor, which could have influenced the results, is age differences in sensory ability (namely eyesight). However, all older dogs in our study were able to pass the criteria in three visual discrimination tasks, and in the geometric forms task, we found no age differences in the number of sessions to criteria (see Supplementary Materials, Table S1). Additionally, we tested many of the subjects in behavioural tests and found little evidence that visual impairments influenced the dogs’ performance (Wallis et al. 2015; Wallis et al. 2014).

The total number of correction trials increased with age in all discrimination tasks possibly due to a lack of attention, persistency and/or side bias in the older dogs, resulting in an inability to adjust thinking or attention in response to feedback. Similarly to earlier findings in dogs (Chan et al. 2002), the oldest age group displayed the most perseverative errors and thus displayed reduced flexibility. Aged members of other species have also shown reduced flexibility reflected in an inability to suppress and/or change behaviour on the basis of negative feedback; for example rats (Stephens et al. 1985), non-human primates (Lai et al. 1995; Manrique and Call 2015; Voytko 1999; Voytko 1993) and humans (Botwinick 1978; Daigneault et al. 1992).

The proportion of test trials in which the dogs chose based on novelty, avoidance or exclusion in test 1 of the inference by exclusion task increased with age. However, no significant differences between the age groups were found. The proportion of test trials in which the dogs chose based on exclusion in test 2 also increased with age, but with most dogs choosing at chance levels. Less than 10 % of dogs in the current study showed patterns of choice consistent with inference by exclusion, indicating that inference by exclusion was not the predominant strategy used by the dogs. In Aust et al.’s (2008) study by comparison, three out of six dogs were found to display this ability.

In contrast to our prediction of a peak in inference by exclusion ability in young adult dogs, seven dogs in middle-to-late adulthood were found to perform above chance, suggesting that they used reasoning by exclusion. Similarly, in non-human primates, one study by Call (2006) found that the ability to reason by exclusion increases with age. Our results are superficially similar to the primate study; however, after looking into the data more carefully, our results seem to reflect a learning rather than a reasoning effect. This learning effect was strongest in younger individuals: In the test trials, the dogs were not rewarded for choosing based on exclusion (choosing S’), which might have made them switch to choosing randomly due to the missing feedback.

A similar effect might explain why in test 1 choosing S’ (based on novelty, avoidance or exclusion) declined from the first to the second cycle. In the tests, younger dogs might have reacted to the lack of feedback sooner/more often than the older dogs, reflecting their more flexible problem solving style. This interpretation is further supported by the impact of the degree of perseverative responding in the training on performance in the inference by exclusion in test 2. After controlling for age, our results indicated that a higher amount of perseverative responding increases the likelihood of finding response patterns consistent with choosing by exclusion. Conversely, the higher degree of flexibility of the younger dogs may have led to a lower probability of choices following the inference by exclusion pattern in this particular paradigm, where test trials were not rewarded. We suggest that older dogs, especially those that were in the more difficult to learn group B, were more likely to stick with their initial choice of S’ due to the fact that they showed greater levels of perseverative responding in the training and consequently had more chance to learn about the negative stimuli. These dogs may have persisted in their choice of S’ in the test trials in test 1, did not alter their strategy in response to the lack of feedback, and may have been able to encode S’ to working memory to enable them to choose S’ when paired with S” a few trials later in test 2. In the study of Aust et al. (2008), all three dogs, which chose by inference by exclusion, and which were also in group B, needed more sessions to reach criteria in the training and therefore had more experience with correction trials, similarly to dogs in our study. Results from studies on aged humans show similar findings of reduced flexibility (shown in difficulties in switching task sets) and deficiencies in adaptation to external feedback (Kray and Lindenberger 2000; Mell et al. 2005), supporting the findings of the current study.

Finally, there was no effect of age or stimulus group on the performance of dogs in the memory test 6 months later. However, the 6-month break was likely too short a time period to enable the detection of age effects. The lack of age effects on long-term memory confirms previous results in laboratory dogs by Araujo et al. (2005). Nearly all the dogs tested in the current study scored above chance in the very first session, suggesting that long-term memory for specific stimuli on the touchscreen is longer than 6 months in dogs. Recently, we re-tested five dogs of different breeds, which had undergone inference by exclusion training between 3 and 5 years previously, and these individuals performed at over 80 % correct first choices on the first day of re-training, which is comparable to the performance of dogs in the memory test of the current study. Therefore, domestic dogs’ long-term memory for picture stimuli may exceed 5 years, similarly to baboons and pigeons (Fagot and Cook 2006).

In conclusion, older dogs showed slower learning and reduced flexibility, which may have contributed to an increase in choosing by inference by exclusion in the tests in comparison to young dogs, which were more sensitive to the lack of feedback in test trials, and subsequently flexibly changed their response pattern and used strategies other than inference by exclusion. Dogs’ long-term memory for the clip art picture discrimination was well maintained into old age. Our results in the visual discrimination learning tasks show clear age differences confirming that the tests used are suitable to detect cognitive aging in pet dogs and provide additional evidence of the suitability of the dog as a model for aging. The baseline measures associated with normal cognitive aging in the pet Border Collie found in the current study can serve as a basis for comparison to help diagnose cognition-related problems and as a tool to assist with the development of treatments to delay cognitive decline. Moreover, the touchscreen apparatus offers a standardised procedure, which can be applied across different dog breeds, other non-human animals and even humans. Utilising this method, future studies could investigate the development and aging of cognitive processes and disorders and their interactions with genetic, environmental and social factors.

## Electronic supplementary material

Below is the link to the electronic supplementary material.

ESM 1 (DOCX 60 kb)
