# Supplementary material for: Aging effects on discrimination learning, logical reasoning and memory in pet dogs
Source: Age (Dordr). 2016 Jan 4;38(1):6. doi: 10.1007/s11357-015-9866-x (PMC5005891; doi:10.1007/s11357-015-9866-x)
Supplement: Supplementary file 1 — (DOCX 60 kb) [file 11357_2015_9866_MOESM1_ESM.docx]

**Supplementary Materials**

Full models are presented for each of the different tasks: geometric forms discrimination, underwater photos and drawings discrimination, clip art picture discrimination, and inferential reasoning by exclusion Test 1 and Test 2. Stepwise removal of non-significant terms in order of decreasing significant was utilised. In case of non-significant effects, the average effect and SE are given at the point of their removal.

Geometric forms discrimination

The geometric form discrimination was run in order for the dogs to become familiar with the two choice discrimination procedure, and to allow dogs to experience for the first time the consequence of touching a negative stimulus (S-). Results indicate that in a simple discrimination of two geometric forms no age differences were detected, however, the dogs had a preference for the ‘*circle’* stimuli, which resulted in a significant difference between the stimulus groups.

Table S1: Negative binomial generalized linear models showing the direction of effects and the significance level of the terms in the geometric forms discrimination.

| Response variable | Full model | Average effect | SE | Wald statistic | P |
| --- | --- | --- | --- | --- | --- |
| Number of sessions to criterion | Stimulus group: Square | 1.2751 | 0.1681 | 46.5038 | **<0.001** |
|  | Sex: Male | 0.2294 | 0.1677 | 1.8688 | 0.1716 |
|  | Age in months | 0.0021 | 0.0025 | 0.6764 | 0.4108 |
|  | Neuter: Neutered | -0.1522 | 0.2082 | 0.5090 | 0.4756 |
|  | Age*Stimulus group | -0.0018 | 0.0050 | 0.1194 | 0.7296 |
| Number of correction trials | Stimulus group: Square | 2.086 | 0.196 | 70.3193 | **<0.001** |
|  | Sex: Male | 0.1966 | 0.2046 | 0.9368 | 0.3331 |
|  | Neuter: Neutered | -0.0945 | 0.2095 | 0.1971 | 0.6571 |
|  | Age in months | 0.00212 | 0.0036 | 0.3024 | 0.5824 |
|  | Age*Stimulus group | -0.0029 | 0.0061 | 0.2127 | 0.6446 |

Table S2: Negative binomial generalized linear models showing the direction of effects and the significance level of the terms in the underwater photos and drawings discrimination. Z tests indicate which age groups differ from age group 1 in the respective analysis.

| Response variable | Model | Minimal model | Average effect | SE | Wald statistic | z | P |
| --- | --- | --- | --- | --- | --- | --- | --- |
| Number of | Model 1 | Stimulus group:Underwater | 1.3841 | 0.1389 | 68.7041 |  | **<0.001** |
| sessions to |  | Age in months | 0.0072 | 0.0018 | 14.2237 |  | **<0.001** |
| Criterion |  | Sex: Male | 0.1566 | 0.1289 | 1.4654 |  | 0.2261 |
|  |  | Neuter: Neutered | -0.1453 | 0.1636 | 0.7659 |  | 0.3815 |
|  |  | Age*Stimulus group | 0.0023 | 0.0037 | 0.3812 |  | 0.5370 |
|  | Model 2 | Stimulus group:Underwater | 1.3841 | 0.1389 | 68.7041 |  | **<0.001** |
|  |  | Age group |  |  | 14.6271 |  | **0.0055** |
|  |  | Age group 2 | 0.0109 | 0.1969 |  | 0.055 | 0.9559 |
|  |  | Age group 3 | 0.1200 | 0.2025 |  | 0.593 | 0.5534 |
|  |  | Age group 4 | 0.4832 | 0.1937 |  | 2.495 | **0.0126** |
|  |  | Age group 5 | 0.6104 | 0.2121 |  | 2.877 | **0.0040** |
| Number of | Model 3 | Stimulus group:Underwater | 1.7887 | 0.1470 | 88.0760 |  | **<0.001** |
| correction |  | Age in months | 0.0067 | 0.0022 | 9.5844 |  | **0.0019** |
| Trials |  | Neuter: Neutered | 0.0866 | 0.1798 | 0.2124 |  | 0.6449 |
|  |  | Sex: Male | -0.0176 | 0.1467 | 0.0142 |  | 0.9050 |
|  |  | Age*Stimulus group | 0.0004 | 0.0045 | 0.0097 |  | 0.9217 |
|  | Model 4 | Stimulus group:Underwater | 1.7887 | 0.1470 | 88.0760 |  | **<0.001** |
|  |  | Age group |  |  | 11.1809 |  | **0.0246** |
|  |  | Age group 2 | -0.0631 | 0.2135 |  | -0.295 | 0.7677 |
|  |  | Age group 3 | 0.3723 | 0.2155 |  | 1.728 | 0.0841 |
|  |  | Age group 4 | 0.4144 | 0.2151 |  | 1.927 | 0.0540 |
|  |  | Age group 5 | 0.5741 | 0.2412 |  | 2.383 | **0.0172** |

Table S3: Negative binomial generalized linear models showing the direction of effects and the significance level of the terms in the inferential reasoning by exclusion training. Z tests indicate which age groups differ from age group 1 in the respective analysis.

| Response variable | Model | Minimal model | Average effect | SE | Wald statistic | z | P |
| --- | --- | --- | --- | --- | --- | --- | --- |
| Number of | Model 5 | Age in months | 0.0100 | 0.0017 | 32.3262 |  | **<0.001** |
| sessions to |  | Sex: Male | 0.3507 | 0.1169 | 8.7099 |  | **0.0032** |
| Criterion |  | Stimulus group: B | 0.2707 | 0.1095 | 5.9078 |  | **0.0151** |
|  |  | Reward ratio: 90% | 0.3486 | 0.1545 | 4.8773 |  | **0.0272** |
|  |  | Age*Stimulus | 0.0035 | 0.0030 | 1.2236 |  | 0.2686 |
|  |  | Neuter: Neutered | 0.1298 | 0.1372 | 0.8820 |  | 0.3476 |
|  | Model 6 | Age group |  |  | 29.6328 |  | **<0.001** |
|  |  | Age group 2 | 0.0612 | 0.2046 |  | 0.2990 | 0.7647 |
|  |  | Age group 3 | 0.1162 | 0.2088 |  | 0.5570 | 0.5778 |
|  |  | Age group 4 | 0.6525 | 0.2193 |  | 2.9750 | **0.0029** |
|  |  | Age group 5 | 0.8879 | 0.2215 |  | 4.0090 | **<0.001** |
| Number of | Model 7 | Age in months | 0.0118 | 0.0019 | 37.9526 |  | **<0.001** |
| correction |  | Stimulus group: B | 0.4313 | 0.1250 | 11.1686 |  | **<0.001** |
| Trials |  | Sex: Male | 0.3184 | 0.1253 | 6.2962 |  | **0.0121** |
|  |  | Neuter: Neutered | 0.2500 | 0.1524 | 2.7396 |  | 0.0979 |
|  |  | Reward ratio: 90% | 0.1845 | 0.1667 | 1.1281 |  | 0.2882 |
|  |  | Age*Stimulus | 0.0038 | 0.0035 | 1.1777 |  | 0.2778 |
|  | Model 8 | Age group |  |  | 32.1295 |  | **<0.001** |
|  |  | Age group 2 | 0.3174 | 0.2287 |  | 1.388 | 0.1652 |
|  |  | Age group 3 | 0.2992 | 0.2338 |  | 1.280 | 0.2007 |
|  |  | Age group 4 | 0.6798 | 0.2490 |  | 2.730 | **0.0063** |
|  |  | Age group 5 | 1.2756 | 0.2525 |  | 5.053 | **<0.001** |

Sex differences

Results from the inference by exclusion training indicate a sex difference in learning ability and rate of perseveration. Male dogs needed more sessions to reach criterion, and more correction trials than females. Sex differences in cognitive abilities are widespread in humans (Andreano & Cahill, 2009; Halpern, 2013; Healy, Bacon, Haggis, Harris, & Kelley, 2009; Mann, Sasanuma, Sakuma, & Masaki, 1990), but sex differences in cognition in areas other than spatial cognition are less well known in non-human mammals. Duranton, Rödel, Bedossa, & Belkhir, (2015) reported differences between male and female dogs in problem solving abilities. Male dogs initially outperformed females, but when successful individuals were retested, females performed better than males. The authors propose that this effect was due to differences in the ability to remember the successful strategy of problem solving, probably due to sex-specific effects on brain differentiation in early life. Also in humans females seem to remember precise object features better than males (Voyer, Postma, Brake, & Imperato-McGinley, 2007). Such a sex difference may help to explain the superior performance of female subjects in our study. Alternatively or additionally, the male dogs' poorer performance in our study could be explained by the fact that male individuals show a greater tendency to perseverate, as seen in humans (Boone, Ghaffarian, Lesser, Hill-Gutierrez, & Berman, 1993; Davis & Nolen-Hoeksema, 2000), rhesus monkeys (Herman & Wallen, 2007), and rats (Guillamón, Valencia, Calés, & Segovia, 1986), suggesting that in some contexts males are cognitively less flexible.

Reward ratio reduction

Thirteen dogs which were tested prior to 2010 were trained on a 100% reward ratio in the inferential reasoning by exclusion training picture discrimination. During the inference by exclusion testing, the dogs experienced unrewarded test trials for the first time. Therefore, their performance may have been influenced by expectancy violation, and they may have been more likely to change their initial choice of stimuli, in response to the fact that the reward was withheld. In effect, they may have interpreted the fact that no food reward was received as negative feedback for an incorrect choice (even though no red screen was presented). Since their performance in the test was likely to have been negatively influenced, for the remaining 72 dogs, the reward ratio in the training was reduced stepwise to allow them to experience unrewarded trials.

Reducing the reward ratio from 100% (as used in Aust et al. (2008)) to 90% resulted in an increase in the number of sessions needed to reach criteria in the training in the current study (see Table S3, Model 5). Previous studies on partial reinforcement and learning rate in humans and animals have discovered that response strength is built up more rapidly when a 100% reinforcement schedule is utilized (Jenkins & Stanley, 1950). However, in the inference by exclusion Test 1, we did not find any effect of partial reinforcement (see Table S4, Model 9). The strong learning effect found from Cycle 1 to Cycle 2 likely overshadowed any positive effects of the reduced reward training.

Since only 3 dogs from the 100% reward group passed Test 1 and went onto Test 2, it was not possible to examine the effect of reward ratio on Test 2. Future studies should aim to develop new methodologies which could allow feedback during test trials to prevent the dogs from switching to a different problem solving strategy.

Stimulus preferences

Stimulus preferences were noted in the geometric forms, the underwater photos and drawings, and inferential reasoning by exclusion training discriminations. Preferences for certain stimulus groups resulted in a decreased number of sessions to criteria, and a decrease in the number of correction trials in comparison to the non-preferred stimulus group. In the geometric forms discrimination we noted that dogs showed a preference for the circle stimuli, in the underwater drawing discrimination, the dogs preferred the drawings over the underwater photographs, and finally in the inferential reasoning by exclusion training, the dogs preferred stimuli in Group ‘*A*’. We can speculate that dogs tend to prefer round stimuli, as many positive objects in their everyday lives are circle shaped (including for instance toys, balls, food bowls, dried dog kibble, and collars). In the inferential reasoning by exclusion training discrimination, the dogs preference for group ‘*A*’ could be due to the fact that in that group there were three stimuli which had a round shape (mug, clock and bowl), compared to only two stimuli in group B (telephone and basket). Dogs preference for the drawings in the underwater photos and drawings discrimination may be explained by a preference for greater contrast in the drawings, and/or an aversion to the comparably darker colouration of the underwater photographs.

Object preferences have been previously documented in laboratory dogs and primates (Adams, Chan, Callahan, & Milgram, 2000; Brush, Mishkin, & Rosvold, 1961). Animals tested in two choice discriminations with their preferred object as positive showed significantly more rapid learning than those tested with their non-preferred object. Using the touchscreen paradigm, O’Hara, Auersperg, Bugnyar, & Huber, (2015) tested inference by exclusion in Goffin’s cockatoos, and found that stimulus preferences was one of several strategies employed by the birds to solve the task. Therefore, preferences for real life objects and two dimensional images on the touchscreen are possible in mammals, and appear to be relatively common during object choice discriminations.

Table S4: Generalized linear mixed model on the proportion of trials chose S’ when paired with a known negative in Test 1, showing the direction of effects and the significance level of the terms. Z tests indicate which age groups differ from age group 1 in the respective analysis.

| Response variable | Model | Minimal model | Average effect | SE | Wald statistic /Deviance | z | P |
| --- | --- | --- | --- | --- | --- | --- | --- |
| Proportion of | Model 9 | Cycle: Cycle 2 | -0.4943 | 0.0839 | 34.723 |  | **<0.001** |
| trials chose S’ |  | Stimulus: Group B | 0.3478 | 0.1007 | 11.136 |  | **<0.001** |
|  |  | Age in months | 0.0037 | 0.0014 | 6.567 |  | **0.0104** |
|  |  | Sex:Male | 0.1919 | 0.0988 | 3.693 |  | 0.0546 |
|  |  | Neuter: Neutered | 0.0953 | 0.1191 | 0.637 |  | 0.4246 |
|  |  | Age*Stimulus | 0.0015 | 0.0028 | 0.299 |  | 0.5845 |
|  |  | Reward ratio: 90% | 0.0562 | 0.1361 | 0.169 |  | 0.6805 |
|  | Model 10 | Age group |  |  | 5.358 |  | 0.2524 |

Table S5: Generalized linear mixed model on the number of times dogs’ chose by inference by exclusion in Test 2, showing the direction of effects and the significance level of the terms. Since there was no significant difference between the number of times dogs’ chose by inference by exclusion in cycle 1 and cycle 2 (model 10), the data was pooled and generalised linear models were applied (Model 10a, 11, 12 and 13). Z tests indicate which age groups differ from age group 1 in the respective analysis.

| Response variable | Model | Minimal model | Average effect | SE | Wald statistic /Deviance | z | P |
| --- | --- | --- | --- | --- | --- | --- | --- |
| Proportion | Model 11 | Cycle | 0.0165 | 0.1279 | 0.016 |  | 0.8977 |
| of times | Model 12 | Age in months | 0.0099 | 0.0014 | 45.538 |  | **<0.001** |
| chose by |  | Stimulus: Group B | 0.7027 | 0.1367 | 27.739 |  | **<0.001** |
| inference by |  | Sex:Male | 0.1112 | 0.1329 | 0.701 |  | 0.4026 |
| exclusion |  | Age*Stimulus | -0.0028 | 0.0032 | 0.765 |  | 0.3819 |
|  |  | Neuter: Neutered | 0.1344 | 0.1771 | 0.573 |  | 0.4490 |
|  | Model 13 | Age group |  |  | 54.570 |  | **<0.001** |
|  |  | Age group 2 | 0.4654 | 0.2816 |  | 1.653 | 0.0984 |
|  |  | Age group 3 | 0.6387 | 0.2989 |  | 2.137 | 0.0326 |
|  |  | Age group 4 | 1.2223 | 0.2900 |  | 4.215 | **<0.001** |
|  |  | Age group 5 | 1.3916 | 0.2788 |  | 4.992 | **<0.001** |
|  |  | Stimulus: Group B | 0.7474 | 0.1413 | 29.420 |  | **<0.001** |
|  | Model 14 | Age in months | 0.0096 | 0.0017 | 45.538 |  | **<0.001** |
|  |  | Sessions to criterion | 0.0008 | 0.0029 | 0.082 |  | 0.7749 |
|  | Model 15 | Age in months | 0.0096 | 0.0017 | 45.538 |  | **<0.001** |
|  |  | Total no. of correction trials | 0.0006 | 0.0003 | 4.103 |  | **0.0428** |

Table S6: Generalized linear model on the proportion of correct trials in the first session of the memory test, showing the direction of effects and the significance level of the terms.

| Response variable | Full model | Average effect | SE | Deviance | P |
| --- | --- | --- | --- | --- | --- |
| Number of correct first choices | Neuter: Neutered | -0.1458 | 0.1397 | 1.0824 | 0.2982 |
| in Session 1 | Age in months | 0.0031 | 0.0030 | 1.0745 | 0.2999 |
|  | Sex: Male | -0.0956 | 0.1509 | 0.3984 | 0.5279 |
|  | Stimulus group: Group B | 0.0562 | 0.1423 | 0.1560 | 0.6929 |
|  | Age*Stimulus group | 0.0043 | 0.0046 | 0.8595 | 0.3539 |
|  |  |  |  |  |  |

References

Adams, B., Chan, A., Callahan, H., & Milgram, N. W. (2000). The canine as a model of human cognitive aging: recent developments. *Progress in Neuro-Psychopharmacology & Biological Psychiatry*, *24*, 675–692.

Andreano, J. M., & Cahill, L. (2009). Sex influences on the neurobiology of learning and memory. *Learning & Memory (Cold Spring Harbor, N.Y.)*, *16*, 248–266. doi:10.1101/lm.918309

Boone, K. B., Ghaffarian, S., Lesser, I. M., Hill-Gutierrez, E., & G. Berman, N. (1993). Wisconsin card sorting test performance in healthy, older adults: Relationship to age, sex, education, and IQ. *Journal of Clinical Psychology*, *49*(1), 54–60. doi:10.1002/1097-4679(199301)49:1<54::AID-JCLP2270490108>3.0.CO;2-6

Brush, E. S., Mishkin, M., & Rosvold, H. E. (1961). Effects of object preferences and aversions on discrimination learning in monkeys with frontal lesions. *Journal of Comparative and Physiological Psychology*, *54*(3), 319–325. doi:10.1037/h0042633

Davis, R. N., & Nolen-Hoeksema, S. (2000). Cognitive inflexibility among ruminators and nonruminators. *Cognitive Therapy and Research*, *24*(6), 699–711. doi:10.1023/A:1005591412406

Duranton, C., Rödel, H. G., Bedossa, T., & Belkhir, S. (2015). Inverse sex effects on performance of domestic dogs (Canis familiaris) in a repeated problem-solving task. *Journal of Comparative Psychology*, *129*, 84–87. doi:10.1037/a0037825

Guillamón, A., Valencia, A., Calés, J. M., & Segovia, S. (1986). Effects of early postnatal gonadal steroids on the successive conditional discrimination reversal learning in the rat. *Physiology & Behavior*, *38*(6), 845–849. doi:10.1016/0031-9384(86)90052-1

Halpern, D. F. (2013). *Sex Differences in Cognitive Abilities: 4th Edition*. Psychology Press. Retrieved from http://books.google.com/books?hl=en&lr=&id=ocl5AgAAQBAJ&pgis=1

Healy, S. D., Bacon, I. E., Haggis, O., Harris, A. P., & Kelley, L. A. (2009). Explanations for variation in cognitive ability: Behavioural ecology meets comparative cognition. *Behavioural Processes*. doi:10.1016/j.beproc.2008.10.002

Herman, R. A., & Wallen, K. (2007). Cognitive performance in rhesus monkeys varies by sex and prenatal androgen exposure. *Hormones and Behavior*, *51*(4), 496–507. doi:10.1016/j.yhbeh.2007.01.005

Jenkins, W. O., & Stanley, J. C. (1950). Partial reinforcement: a review and critique. *Psychological Bulletin*, *47*, 193–234.

Mann, V. A., Sasanuma, S., Sakuma, N., & Masaki, S. (1990). Sex differences in cognitive abilities: a cross-cultural perspective. *Neuropsychologia*, *28*(10), 1063–1077. doi:10.1016/0028-3932(90)90141-A

O’Hara, M., Auersperg, A. M. I., Bugnyar, T., & Huber, L. (2015). Inference by Exclusion in Goffin Cockatoos (Cacatua goffini). *PLOS ONE*, *10*(8), e0134894. doi:10.1371/journal.pone.0134894

Voyer, D., Postma, A., Brake, B., & Imperato-McGinley, J. (2007). Gender differences in object location memory: a meta-analysis. *Psychonomic Bulletin & Review*, *14*(1), 23–38. doi:10.3758/BF03194024
